# Specificity in Rehabilitation of Word Production: A Meta-Analysis and a Case Study

**DOI:** 10.3233/BEN-2012-0358

**Published:** 2012-03-16

**Authors:** Charlotte Jacquemot, Emmanuel Dupoux, Laura Robotham, Anne-Catherine Bachoud-Lévi

**Affiliations:** ^1^Laboratoire de Sciences Cognitives et PsycholinguistiqueENS-EHESS-CNRSParisFrance; ^2^Département d’Etudes CognitivesEcole Normale SupérieureParisFrance; ^3^INSERM U955 team 1 “Neuropsychologie Interventionnelle”Institut Mondor de Recherche Biomédicale CréteilFrance; ^4^AP/HPUnité de NeuropsychologieService de NeurologieHôpital Henri MondorCréteilFrance; ^5^Université Paris 12Faculté de MédecineCréteilFrance

**Keywords:** Word production, aphasia, rehabilitation, model-driven approach, meta-analysis, case study

## Abstract

Speech production impairment is a frequent deficit observed in aphasic patients and rehabilitation programs have been extensively developed. Nevertheless, there is still no agreement on the type of rehabilitation that yields the most successful outcomes. Here, we ran a detailed meta-analysis of 39 studies of word production rehabilitation involving 124 patients. We used a model-driven approach for analyzing each rehabilitation task by identifying which levels of our model each task tapped into. We found that (1) all rehabilitation tasks are not equally efficient and the most efficient ones involved the activation of the two levels of the word production system: the phonological output lexicon and the phonological output, and (2) the activation of the speech perception system as it occurs in many tasks used in rehabilitation is not successful in rehabilitating word production. In this meta-analysis, the effect of the activation of the phonological output lexicon and the phonological output cannot be assessed separately. We further conducted a rehabilitation study with DPI, a patient who suffers from a damage of the phonological output lexicon. Our results confirm that rehabilitation is more efficient, in terms of time and performance, when specifically addressing the impaired level of word production.

## 1. Introduction

Production difficulties are reported to be the most frustrating and distressing aspects of aphasia and one of the first causes of social drop out and depression among patients [1]. After an initial phase of spontaneous recovery, the speech performance of aphasic patients usually stabilizes, but sometimes considerably below the unimpaired level. Restoring language production through rehabilitation is therefore of considerable clinical interest. Yet, there is still considerable disagreement regarding the most efficient rehabilitation strategy, and it is still unclear whether it should target the language faculty broadly or focus on the specific disorder at hand. In fact, several authors have claimed after years of research that no clear connection between the type of impairment and the most effective therapy can be established (see for instance [2–4]). In this paper, we argue that such questions can be clarified by relying on a single language processing model used simultaneously for the diagnosis of the deficit and for the design of rehabilitation tasks. We illustrate this point with a statistical meta-analysis of 39 rehabilitation studies, and a case study of the rehabilitation of an aphasic patient.

One of the obstacles in comparing the outcome of rehabilitation studies lies in their different mix of train-

ISSN 0953-4180/12/$27.50 © 2012 – IOS Press and the authors. All rights reserved

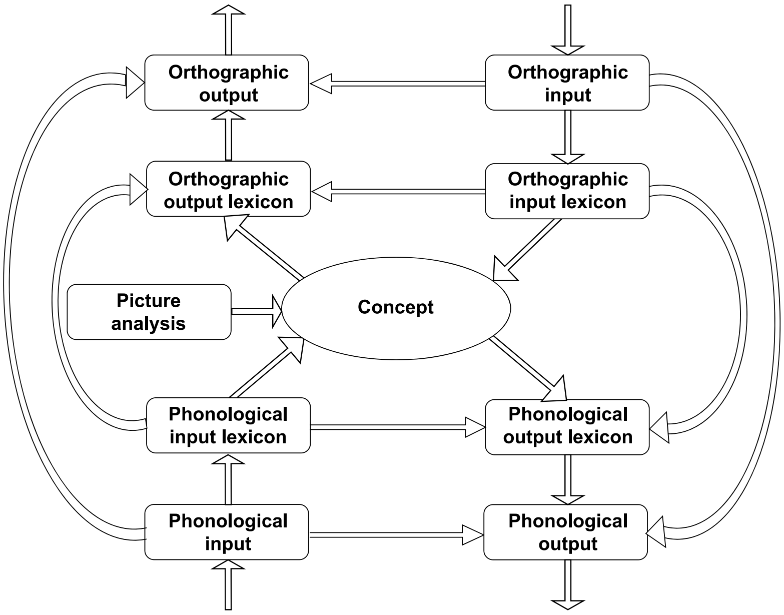

Fig. 1. Model of language processing including speech input and output systems and written input and output systems.

ing tasks. One can distinguish two broad types of therapies: so-called semantic and phonological therapies. Semantic therapy includes tasks such as word-picture matching, naming on definition, and questions on semantic features of a picture. Phonological therapy includes tasks such as word reading, word repetition, picture naming with phonological cueing, and tasks that require monitoring word features (number of syllables, first phoneme or first syllable) on an implicitly named picture or from an auditory input. These two therapies were proposed to correspond to two broad classes of production deficits: respectively, semantic therapy for patients who make semantic errors in selecting words during production, and phonological therapy for patients who select the right words, but make phonological errors during planning [5–12]. However, such a selective effect of rehabilitation strategy is challenged by the observation of rather indiscriminate effects of therapy as a function of deficit [13–17]. Marshall and collaborators [13] observed a positive effect of semantic rehabilitation in patient with no semantic deficit while Nickels and Best [16] described a patient suffering from semantic impairment who failed to benefit from therapy involving semantic tasks. Additionally, phonological tasks have also been successful in improving word production abilities in patients with semantic impairment [10,11,14,15,18–20]. Finally, the absence of rehabilitation effects whatever the strategy, whether it be semantic or phonologic, is reported in some but very few studies [4,11,21–23].

A similarly confusing pattern of results is found regarding the existence of generalization of the rehabilitation results beyond the set of specifically trained items. Some studies find generalization [12,14–16,18, 24–26], whereas some studies failed to observe it [5, 7,10,13–15,17,25–27]. Hillis [7] proposed that unlike phonological therapy, semantic therapy could induce generalization for untrained items that are semantically related to the trained items. However, generalization has also been obtained with phonological therapy [12].

Overall, from these studies, it appears that phonological and semantic therapies are both potentially useful for aphasic rehabilitation, but that relationships between impairment and rehabilitation tasks are not straightforward [6]. One reason could be that there is no relationship to be found: language rehabilitation requires the activation of the whole language network, without regard for the particular deficit. If this is true, there is no point in trying to devise therapies tailored to a particular language deficit. However, before endorsing such a strong conclusion, one needs to inspect in close detail the possibility that the tasks used in the different kinds of therapies are not very pure and involve overlapping processing levels. We illustrate this point with the model presented in Fig. 1. This is a fairly standard functional architecture for language processing at the level of individual words adapted from Miceli and colleagues [28], (see also, [29,30]). It distinguished two input modalities: one for spoken words and one for written words. Similarly, it recognized two output modalities. Within each modality there is a formal level (orthography or phonology) and a lexical level. The lexical levels are connected to a common a-modal conceptual level, and connected to each other. The formal levels are connected to perceptual inputs or motor outputs and to their respective lexical levels. Finally, some formal levels are connected to each other across modalities (i.e. input phonology to output phonology). Within such a model, any psycholinguistic task can be analyzed as involving a particular pattern of levels and connections: for instance, a picture naming task involves the following pathway: Picture analysis – Conceptual level – Output Phonological Lexicon – Output Phonology.

With such a model in mind, a quick inspection of some of the tasks used in rehabilitation studies reveals that in many if not in all 'semantic tasks', the form of the word is provided as a spoken or written word, or as a feedback response of the experimenter [5,7,13,14, 16]. This implies that these semantic tasks are activating phonological and/or orthographic levels. Vice versa, many 'phonological tasks' involve real words presented auditorily or orthographically, which therefore activate lexical and probably also semantic levels. As Byng [31] explicitly states, "most therapies described do not represent a single therapeutic process, even if they involve a single task. A single task might require a number of complex processes, but it may not be clear which of these processes was the most important for affecting change" (p.10). Since the rehabilitation tasks do not isolate distinct levels of processing, it is therefore not so surprising that they do not appear to be very specific.

In this paper, we propose to revisit the issue of rehabilitation specificity by relying on the processing model presented in Fig. 1. We first reanalyzed the tasks used in 39 rehabilitation studies of patients with naming deficits. This analysis was done by identifying for each task the levels of the model stimulated and connections they activated. We then derived a measure of therapeutic sensitivity for each of these levels and connections, and showed that rehabilitation is much more specific than the authors of the studies themselves concluded. Secondly, we constructed a rehabilitation protocol with 'pure tasks', that is, tasks that are much more specific to a particular subpart of the language processing system than usually used, and evaluate the effectiveness of four pure rehabilitation procedures on a single anomic patient. We conclude by discussing the value of using a processing model for devising rehabilitation strategies.

## 2. Meta-analysis of rehabilitation of naming

### *2.1. Search strategy and selection criteria*

First, we searched articles in the PubMed and Web of Science databases between 1990 and 2009 (November) using the following key words: speech production, therapy, anomia, rehabilitation, and aphasic patient. We expanded this base by looking up the references cited in the identified articles, arriving at a total of 85 studies. Second, we selected the papers that met the following five criteria:

(1) specific focus on word production rehabilitation,
(2) detailed description of material and of the rehabilitation tasks allowing us to infer the specific processes that were trained,
(3) availability of individual results when the rehabilitation program was proposed to a group of patients,
(4) report of performance baseline before rehabilitation,
(5) report of the rehabilitation outcome, at least qualitatively.

According to these inclusion criteria, 39 speech production rehabilitation studies were selected to be included in our meta-analysis (see Table 1). The reasons of article exclusion were the following: not a word rehabilitation study ($N = 31$), no individual data ($N = 6$), no details concerning the therapy used ($N = 1$), patient with high variability of performance during pretest ($N = 2$), no access to the articles ($N = 6$). Two separate investigators did the reviewing phase and the inclusion phase. Overall, these studies represent a total of 124 individual patient rehabilitation results (some studies have several patients, and some patients are subject to several rehabilitation procedures). Despite this selection, two difficulties remain which hamper the use of the standard rules of meta-analysis involving computing an overall meta-effect size. First, many papers did not report effect sizes, and some of them were even lacking numerical results. Second, because the performance under scrutiny is word production (which implies a difficult to assess "chance" level for performance), and that the lists of words used for test vary widely across studies, it is very difficult to compare the relative strength of rehabilitation between studies (control subjects being at ceiling).

Thus, we resorted to the use of a binary criterion of rehabilitation success (success vs. failure), and the signal detection theory in order to tease apart the un-

Table 1
Word production rehabilitation studies included in the meta-analysis

| Paper | Patient | Treatment task | Rehabilitation outcome |
|---|---|---|---|
| [72] | BR | Picture naming | + |
| | | Repetition | |
| | | Picture naming with phonological cues (letter, syllable, word) and distractors words | |
| | | Elicit rhymes, initial and final phonemes, number of syllables on picture | |
| | | Picture naming with semantic cues | |
| | | Naming to auditory definition | |
| | JD | Repetition | + |
| | | Picture naming with phonological cues (letter, syllable, word) and distractors words | |
| | | Elicit rhymes, initial and final phonemes, number of syllables on picture | |
| | | Picture naming with semantic cues | |
| | | Naming to auditory definition | |
| | | Repetition | |
| | KS | Repetition | + |
| | | Picture naming with phonological cues (letter, syllable, word) and distractors words | |
| | | Elicit rhymes, initial and final phonemes, number of syllables on picture | |
| | | Picture naming with semantic cues | |
| | | Naming to auditory definition | |
| | | Repetition | |
| | BB | Picture naming | + |
| | | Repetition | |
| | | Picture naming with phonological cues (letter, syllable, word) and distractors words | |
| | | Elicit rhymes, initial and final phonemes, number of syllables on picture | |
| | | Picture naming with semantic cues | |
| | | Naming to auditory definition | |
| | HF | Picture naming | + |
| | | Repetition | |
| | | Picture naming with phonological cues (letter, syllable, word) and distractors words | |
| | | Elicit rhymes, initial and final phonemes, number of syllables on picture | |
| | | Picture naming with semantic cues | |
| | | Naming to auditory definition | |
| | WM | Picture naming | + |
| | | Repetition | |
| | | Picture naming with phonological cues (letter, syllable, word) and distractors words | |
| | | Elicit rhymes, initial and final phonemes, number of syllables on picture | |
| | | Picture naming with semantic cues | |
| | | Naming to auditory definition | |
| [42] | RF | Picture naming with orthographic cues | + |
| | | Repetition | − |
| | | Reading aloud | + |
| | MR | Picture naming with orthographic cues | + |
| | | Repetition | − |
| | | Reading aloud | + |
| [45] | JOW | Written naming | − |
| | | Delayed copy | |
| | | Auditory word picture matching | + |
| | | Delayed copying | |
| [73] | 02-030 | Picture naming | + |
| | | Auditory perception of the correct picture name | |
| | | Repetition | |
| | 03-031 | Picture naming | + |
| | | Auditory perception of the correct picture name | |
| | | Repetition | |
| | 00-008 | Picture naming | + |
| | | Auditory perception of the correct picture name | |
| | | Repetition | |

Table 1, continued

| Paper | Patient | Treatment task | Rehabilitation outcome |
|---|---|---|---|
| | 02-036 | Picture naming | + |
| | | Auditory perception of the correct picture name | |
| | | Repetition | |
| | 03-004 | Picture naming | − |
| | | Auditory perception of the correct picture name | |
| | | Repetition | |
| [47] | MB | Reading aloud | + |
| | | Repetition | |
| | | Picture naming with semantic cues | |
| | | Picture naming with phonological cues | |
| | | Auditory word picture matching | |
| | NO | Reading aloud | + |
| | | Repetition | |
| | | Picture naming with semantic cues | |
| | | Picture naming with phonological cues | |
| | | Auditory word picture matching | |
| | PU | Reading aloud | + |
| | | Repetition | |
| | | Picture naming with semantic cues | |
| | | Picture naming with phonological cues | |
| | | Auditory word picture matching | |
| | SK | Reading aloud | + |
| | | Repetition | |
| | | Picture naming with semantic cues | |
| | | Picture naming with phonological cues | |
| | | Auditory word picture matching | |
| [48] | GM | Picture naming with phonological cues | + |
| | | Picture naming with orthographic cues | |
| | | Auditory sentence completion oral and written response | |
| | AS | Picture naming with phonological cues | + |
| | | Picture naming with orthographic cues | |
| | | Auditory sentence completion oral and written response | |
| | BM | Picture naming with phonological cues | + |
| | | Picture naming with orthographic cues | |
| | | Auditory sentence completion oral and written response | |
| | EL | Picture naming with phonological cues | + |
| | | Picture naming with orthographic cues | |
| | | Auditory sentence completion oral and written response | |
| | EG | Picture naming with phonological cues | + |
| | | Picture naming with orthographic cues | |
| | | Auditory sentence completion oral and written response | |
| | RH | Picture naming with phonological cues | + |
| | | Picture naming with orthographic cues | |
| | | Auditory sentence completion oral and written response | |
| [74] | TV | Picture naming | + |
| | | Repetition | |
| | | Picture naming with phonological cues (letter, syllable, word) and distractors words | |
| | | Elicit rhymes, initial and final phonemes, number of syllables on picture | |
| | | Picture naming with semantic cues | |
| | | Naming to auditory definition | |
| [18] | MB | Word length judgment on auditory words | + |
| | | Spoken-to-written phoneme matching | |
| | | Point to initial/final letter of auditory words | |
| | | Point to written word that rhymes with auditory word | |
| | | Auditory word picture matching | + |
| | | Auditory word picture matching + picture naming task | |
| | | Picture naming with error judgment | |

Table 1, continued

| Paper | Patient | Treatment task | Rehabilitation outcome |
|---|---|---|---|
| [75] | NS | Picture naming with phonological cues (letter, syllable, word) and distractors words | – |
| | | Repetition | |
| | | Picture naming with semantic cues | – |
| | | Repetition | |
| | EG | Picture naming with phonological cues (letter, syllable, word) and distractors words | + |
| | | Repetition | |
| | | Picture naming with semantic cues | + |
| | | Repetition | |
| | CH | Picture naming with phonological cues (letter, syllable, word) and distractors words | + |
| | | Repetition | |
| | | Picture naming with semantic cues | + |
| | | Repetition | |
| [5] | SS | Naming to auditory definition | + |
| | | Repetition | |
| | | Picture naming with semantic cues | + |
| | | Elicit category, visual features, size from picture | |
| | | Repetition | |
| | | Repetition with picture | – |
| | MR | Naming to auditory definition | + |
| | | Repetition | |
| | | Picture naming with visual semantic cues | + |
| | | Elicit category, visual features, size from picture | |
| | | Repetition | |
| | | Repetition with picture | – |
| [49] | HM | Picture naming with orthographic cues letter, syllable, word and distractors words | + |
| | | Repetition | |
| | | Picture naming with phonological cues letter, syllable, word and distractors words | |
| | PH | Picture naming with orthographic cues letter, syllable, word and distractors words | + |
| | | Repetition | |
| | | Picture naming with phonological cues letter, syllable, word and distractors words | |
| | SC | Picture naming with orthographic cues letter, syllable, word and distractors words | – |
| | | Repetition | |
| | | Picture naming with phonological cues letter, syllable, word and distractors words | |
| | DC | Picture naming with orthographic cues letter, syllable, word and distractors words | + |
| | | Repetition | |
| | | Picture naming with phonological cues letter, syllable, word and distractors words | |
| | OL | Picture naming with orthographic cues letter, syllable, word and distractors words | + |
| | | Repetition | |
| | | Picture naming with phonological cues letter, syllable, word and distractors words | |
| | IK | Picture naming with orthographic cues letter, syllable, word and distractors words | + |
| | | Repetition | |
| | | Picture naming with phonological cues letter, syllable, word and distractors words | |
| | NK | Picture naming with orthographic cues letter, syllable, word and distractors words | + |
| | | Repetition | |
| | | Picture naming with phonological cues letter, syllable, word and distractors words | |
| | KR | Picture naming with orthographic cues letter, syllable, word and distractors words | + |
| | | Repetition | |
| | | Picture naming with phonological cues letter, syllable, word and distractors words | |
| [50] | MH | Picture naming with orthographic cues letter, syllable, word and distractors words | + |
| | | Repetition | |
| | | Picture naming with phonological cues letter, syllable, word and distractors words | |
| | HP | Picture naming with orthographic cues letter, syllable, word and distractors words | + |
| | | Repetition | |
| | | Picture naming with phonological cues letter, syllable, word and distractors words | |
| [25] | JJ | Auditory word picture matching | + |
| | | Reading aloud | – |

Table 1, continued

| Paper | Patient | Treatment task | Rehabilitation outcome |
|---|---|---|---|
| | HW | Auditory word picture matching | − |
| | | Reading aloud | + |
| | | Picture naming | + |
| | | Auditory sentence completion oral response | |
| | | Picture naming with phonological cues | |
| | | Repetition | |
| | KE | Picture naming | + |
| | | Auditory sentence completion oral response | |
| | | Picture naming with phonological cues | |
| | | Repetition | |
| [7] | HG | Written picture naming with anagram, orthographic cues | + |
| | | Writing to dictation | |
| | | Delayed copy | |
| | | Picture naming | + |
| | | Written picture naming | |
| | | Reading aloud | |
| | | Question about function of object pictures | |
| | | Auditory sentence completion oral response | |
| | | Picture naming with phonological cues | |
| [40]; [3,45,76] | JOW | Point to initial letter of auditory words | + |
| | | Picture naming with phonological cues | |
| | | Picture naming | |
| | | Repetition | |
| [41] | PD | Repetition | − |
| | | Reading aloud | + |
| | | Auditory sentence completion oral response | − |
| [77] | PG | Written naming | + |
| | | Reading | |
| | AH | Written naming | + |
| | | Reading | |
| | TM | Written naming | + |
| | | Reading | |
| [8] | LR | Auditory word picture matching | + |
| | | Listening oral definition | |
| | | Picture naming with orthographic cues | |
| | | Reading aloud | |
| [43] | P1 | Elicit rhymes, initial and final phonemes, number of syllables on picture | + |
| | | Picture naming | |
| | | Repetition with picture | |
| | P2 | Elicit rhymes, initial and final phonemes, number of syllables on picture | + |
| | | Picture naming | |
| | | Repetition with picture | |
| | P3 | Elicit rhymes, initial and final phonemes, number of syllables on picture | + |
| | | Picture naming | |
| | | Repetition with picture | |
| | P4 | Elicit rhymes, initial and final phonemes, number of syllables on picture | + |
| | | Picture naming | |
| | | Repetition with picture | |
| | P5 | Elicit rhymes, initial and final phonemes, number of syllables on picture | + |
| | | Picture naming | |
| | | Repetition with picture | |
| | P6 | Elicit rhymes, initial and final phonemes, number of syllables on picture | + |
| | | Picture naming | |
| | | Repetition with picture | |

Table 1, continued

| Paper | Patient | Treatment task | Rehabilitation outcome |
|---|---|---|---|
| | P7 | Elicit rhymes, initial and final phonemes, number of syllables on picture<br>Picture naming<br>Repetition with picture | – |
| | P8 | Elicit rhymes, initial and final phonemes, number of syllables on picture<br>Picture naming<br>Repetition with picture | + |
| | P9 | Elicit rhymes, initial and final phonemes, number of syllables on picture<br>Picture naming<br>Repetition with picture | – |
| | P10 | Elicit rhymes, initial and final phonemes, number of syllables on picture<br>Picture naming<br>Repetition with picture | – |
| [21] | BB | Written generation of semantic cues of picture<br>Naming<br>Reading aloud | + |
| | BG | Written generation of semantic cues of picture<br>Naming<br>Reading aloud | + |
| | SB | Written generation of semantic cues of picture<br>Naming<br>Reading aloud | + |
| [13] | RS | Written word picture matching with reading aloud | + |
| | IS | Written word picture matching with reading aloud | + |
| | RS | Written word picture matching with reading aloud | + |
| [78] | CM | Association of picture with semantically related written word<br>Written word picture matching | – |
| [10] | RBO | Reading aloud<br>Picture naming | + |
| | | Repetition<br>Picture naming | + |
| | GMA | Reading aloud with picture<br>Picture naming | + |
| | | Reading aloud<br>Picture naming | + |
| | | Repetition<br>Picture naming with phonologic cues | + |
| [79] | HR | Written naming with anagram, orthographic cues<br>Copy | + |
| [14] | TRC | Written word picture matching | + |
| | | Auditory word picture matching | – |
| | PA | Auditory word picture matching | – |
| | | Reading aloud | + |
| | | Picture naming with orthographic cue and reading aloud | + |
| [80] | WT | Repetition | + |
| | | Repetition with iconic gesture | + |
| [51] | KB | Simultaneous auditory and written word-picture matching<br>Auditory word picture matching<br>Picture naming with phonological or orthographic cue<br>Copy, anagram<br>Written picture naming | + |
| | JI | Simultaneous auditory and written word-picture matching<br>Auditory word picture matching<br>Picture naming with phonological or orthographic cue<br>Copy, anagram<br>Written picture naming | + |

Table 1, continued

| Paper | Patient | Treatment task | Rehabilitation outcome |
|---|---|---|---|
| | RI | Simultaneous auditory and written word-picture matching | + |
| | | Auditory word picture matching | |
| | | Picture naming with phonological or orthographic cue | |
| | | Copy, anagram | |
| | | Written picture naming | |
| [20] | WR | Picture naming | + |
| | | Elicit initial phoneme and rhyme judgement on auditory word | |
| | | Repetition | |
| | | Picture naming | + |
| | | Semantic judgement on auditory word | |
| | | Repetition | |
| | | Repetition with picture | + |
| | | Auditory word picture matching | + |
| [15,26] | CG | Picture naming with phonological cues | + |
| | | Repetition | |
| | RJ | Picture naming with phonological cues | + |
| | | Repetition | |
| [81] | YK | Picture naming | + |
| | | Repetition | |
| | | Reading | |
| [82] | LV | Picture naming | + |
| | | Written word picture matching with reading aloud | |
| | | Repetition | |
| | | Auditory to written word matching | |
| | JP | Picture naming | + |
| | | Written word picture matching with reading aloud | |
| | | Repetition | |
| | | Auditory to written word matching | |
| [83] | P1 | Elicit category, visual features, size from picture | + |
| | | Picture naming | |
| | | Picture naming with semantic cues | |
| | P2 | Elicit category, visual features, size from picture | + |
| | | Picture naming | |
| | | Picture naming with semantic cues | |
| | P3 | Elicit category, visual features, size from picture | + |
| | | Picture naming | |
| | | Picture naming with semantic cues | |
| [12] | GF | Syllable judgement on picture name supplied with the picture | + |
| | | Initial phoneme judgement/pointing on picture name supplied with the picture | |
| | | Naming | |
| [84] | JB | picture naming and iconic gesture of the target | + |
| | | picture naming | + |
| | | iconic gesture | + |
| [52] | AB | Picture naming with syllable counting and first phoneme, identification of picture naming errors | + |
| | | Written anagram and reading | |
| | | Picture naming | |
| | | Repetition | |
| | | Picture naming with syllables counting and iconic gesture of the target | + |
| | | Repetition | |
| | | Picture naming with syllables counting and iconic gesture of the target | + |
| | | Written anagram and reading | |
| | | Repetition | |
| [85] | Anomic patient | Auditory sentence completion oral response | + |
| | | Auditory sentence completion with semantic cues oral response | |
| | | Auditory sentence completion with phonological cues oral response | |
| | | Repetition | |

derlying effects of rehabilitation from the intrinsic biases in relation to the decision threshold for success or failure left to the experimenter (or the reviewers of the study). Although this constitutes a departure from the standard meta-analysis, it shares with it the same general methodology and aims. As can be seen in Table 1, the number of case studies in which the rehabilitation benefited to the patients vastly outnumbers case studies where rehabilitation did not ($N = 105$ vs. $N = 19$). This suggests that studies mostly selected a successful rehabilitation, or that any kind of rehabilitation works (placebo effect), or a reporting bias (that is, unsuccessful studies tend to have more difficulty reaching the publication stage). In addition, some treatments are more frequently used for rehabilitating patients, as for instance naming tasks compared to writing tasks, and therefore some levels are more frequently involved during rehabilitation. In order to rule out any frequency effect of the task, as well as the imbalance between success and failure rates in our meta-analysis, we carried out a signal detection theoretic analysis aimed at distinguishing rehabilitation sensitivity (i.e., the selective effect of rehabilitation) from response bias.

To this end, we used a strategy similar to the ones used by Indefrey and Levelt in his meta-analysis of the functional brain imagery of speech production [32]. First, we assigned to each rehabilitation task a particular "activation pattern" deriving from the framework of the model in Fig. 1. The activation pattern constitutes all components of the model involved in each rehabilitation task. Second, we correlated the activation pattern across studies with rehabilitation outcome for each patient. This allowed us to derive, using signal detection theory, a measure of rehabilitation sensitivity showing component by component its role in rehabilitation outcome.

## 2.2. Method

### 2.2.1. Task modeling

In order to perform a given task, (picture naming, word repetition, etc.) certain components of the model presented in Fig. 1 are essential, and others not. We applied the standard task decomposition method of cognitive neuropsychology [33–35] for each task to identify its functional components according to the model of Fig. 1: we determined a "pattern of activation" by assigning for each task the value "0" or "1" to the processing levels and their connections: an activation value of 1 was assigned to all levels and connections involved in the task, and an activation value of zero otherwise (see Table 2).

The pattern of activation for a given task was determined in order to reflect the main pathway or pathways that subjects use to achieve correct performance in that task. More specifically, we first identified the most direct processing route for each task, which yielded a set of processing levels and connections. In addition, we added less direct routes, if existing psycholinguistic evidence documented that they were generally being used for this task. For instance, the most direct pathway for a picture naming task is that from picture analysis to conceptual level, phonological output lexicon and phonological output. Of course, it is theoretically possible to name a picture using a more complicated route, for instance by accessing the orthographic lexicon and then orthography to phonology, however, since it is not the most straightforward route, and this pathway has not been documented by psycholinguistic investigations, this was not considered. Therefore the activation pattern for picture naming was set to include all of the said levels as well as their connections to each other in the model. For the word repetition task, however, we modeled three pathways, the input-to-output phonology pathway, the input lexicon-to-output lexicon pathway, and the pathways going through the conceptual level. This is because there is evidence that these three pathways are used by participants [29, 36–38]. Two investigators (CJ and ED) independently computed the pattern of activation for each task and a third investigator (ACBL) checked each pattern.

### 2.2.2. Data analysis

As shown in Table 1, we recoded each rehabilitation case study in terms of success (+) or failure (−), based on the data available in the original papers. Success was attributed to studies in which speech production performance improved after treatment, and failure attributed to studies in which speech production did not improved after the treatment, according to the statistical analyses, when available, or to the author's conclusions when not. We then conducted a signal detection theory analysis for each component of the processing model, by considering the signal to be the activation of that component in the rehabilitation task, and the response being whether the rehabilitation was reported to be a success or a failure. For each rehabilitation case study, if the processing component had an activation of 1 for one of the rehabilitation tasks, we scored a HIT if the rehabilitation was successful and a MISS otherwise. If the processing component was not activated in any of

Table 2

Pattern of activation for the different tasks used in word production rehabilitation PA: Picture Analysis, C: Concept; PI: Phonological Input; PIL: Phonological Input Lexicon; POL: Phonological Output Lexicon; PO: Phonological Output; OI: Orthographic Input; OIL: Orthographic Input Lexicon; OOL: Orthographic Output Lexicon; OO: Orthographic Output

| | PA-C | PI-PIL | PIL-C | C-POL | POL-PO | OI-OIL | OIL-C | C-OOL | OOL-OO | OI-PO | OIL-POL | OIL-OOL | OI-OO | PI-PO | PI-OO | PIL-POL | PIL-OOL | PA | C | OOL | OO | POL | PO | PI | PIL | OI | OIL |
|---|---|---|---|---|---|---|---|---|---|---|---|---|---|---|---|---|---|---|---|---|---|---|---|---|---|---|---|
| Association of picture with semantically related written word | 1 | 0 | 0 | 0 | 0 | 1 | 1 | 0 | 0 | 0 | 0 | 0 | 0 | 0 | 0 | 0 | 0 | 1 | 1 | 0 | 0 | 0 | 0 | 0 | 0 | 1 | 1 |
| Auditory perception of the correct picture name | 1 | 1 | 1 | 0 | 0 | 0 | 0 | 0 | 0 | 0 | 0 | 0 | 0 | 0 | 0 | 0 | 0 | 1 | 1 | 0 | 0 | 0 | 0 | 1 | 1 | 0 | 0 |
| Auditory sentence completion (oral and written response) | 0 | 1 | 1 | 1 | 1 | 0 | 0 | 1 | 1 | 0 | 0 | 0 | 0 | 0 | 0 | 0 | 0 | 0 | 1 | 1 | 1 | 1 | 1 | 1 | 1 | 0 | 0 |
| Auditory sentence completion (oral response) | 0 | 1 | 1 | 1 | 1 | 0 | 0 | 0 | 0 | 0 | 0 | 0 | 0 | 0 | 0 | 0 | 0 | 0 | 1 | 0 | 0 | 1 | 1 | 1 | 1 | 0 | 0 |
| Auditory sentence completion with phonological cues (oral response) | 0 | 1 | 1 | 1 | 1 | 0 | 0 | 0 | 0 | 0 | 0 | 0 | 0 | 1 | 0 | 0 | 0 | 0 | 1 | 0 | 0 | 1 | 1 | 1 | 1 | 0 | 0 |
| Auditory sentence completion with semantic cues (oral response) | 0 | 1 | 1 | 1 | 1 | 0 | 0 | 0 | 0 | 0 | 0 | 0 | 0 | 0 | 0 | 0 | 0 | 0 | 1 | 0 | 0 | 1 | 1 | 1 | 1 | 0 | 0 |
| Auditory word picture matching | 1 | 1 | 1 | 0 | 0 | 0 | 0 | 0 | 0 | 0 | 0 | 0 | 0 | 0 | 0 | 0 | 0 | 1 | 1 | 0 | 0 | 0 | 0 | 1 | 1 | 0 | 0 |
| Auditory word – written word matching | 0 | 1 | 1 | 1 | 1 | 1 | 1 | 0 | 0 | 1 | 1 | 0 | 0 | 1 | 0 | 1 | 0 | 0 | 1 | 0 | 0 | 1 | 1 | 1 | 1 | 1 | 1 |
| Auditory word picture matching + picture naming | 1 | 1 | 1 | 1 | 1 | 0 | 0 | 0 | 0 | 0 | 0 | 0 | 0 | 0 | 0 | 0 | 0 | 1 | 1 | 0 | 0 | 1 | 1 | 1 | 1 | 0 | 0 |
| Copy | 0 | 0 | 0 | 0 | 0 | 1 | 1 | 1 | 1 | 0 | 0 | 1 | 1 | 0 | 0 | 0 | 0 | 0 | 1 | 1 | 1 | 0 | 0 | 0 | 0 | 1 | 1 |
| Copy, anagram | 0 | 0 | 0 | 0 | 0 | 1 | 1 | 1 | 1 | 0 | 0 | 1 | 1 | 0 | 0 | 0 | 0 | 0 | 1 | 1 | 1 | 0 | 0 | 0 | 0 | 1 | 1 |
| Delayed copy | 0 | 0 | 0 | 0 | 0 | 1 | 1 | 1 | 1 | 0 | 0 | 1 | 1 | 0 | 0 | 0 | 0 | 0 | 1 | 1 | 1 | 0 | 0 | 0 | 0 | 1 | 1 |
| Elicit category, visual features, size from picture | 1 | 0 | 0 | 1 | 1 | 0 | 0 | 0 | 0 | 0 | 0 | 0 | 0 | 0 | 0 | 0 | 0 | 1 | 1 | 0 | 0 | 1 | 1 | 0 | 0 | 0 | 0 |
| Elicit initial phoneme and rhyme judgement on auditory word | 0 | 1 | 1 | 0 | 0 | 0 | 0 | 0 | 0 | 0 | 0 | 0 | 0 | 0 | 0 | 0 | 0 | 1 | 1 | 0 | 0 | 0 | 0 | 1 | 1 | 0 | 0 |
| Elicit rhymes, initial and final phonemes, number of syllables of picture name | 1 | 0 | 0 | 1 | 1 | 0 | 0 | 0 | 0 | 0 | 0 | 0 | 0 | 0 | 0 | 0 | 0 | 1 | 1 | 0 | 0 | 1 | 1 | 0 | 0 | 0 | 0 |

Table 2, continued

| | PA-C | PI-PIL | PIL-C | C-POL | POL-PO | OI-OIL | OIL-C | C-OOL | OOL-OO | OI-PO | OIL-POL | OIL-OOL | OI-OO | PI-PO | PI-OO | PIL-POL | PIL-OOL | PA | C | OOL | OO | POL | PO | PI | PIL | OI | OIL |
|---|---|---|---|---|---|---|---|---|---|---|---|---|---|---|---|---|---|---|---|---|---|---|---|---|---|---|---|
| Initial phoneme judgement /pointing on picture name supplied with the picture | 1 | 1 | 1 | 0 | 0 | 0 | 0 | 0 | 0 | 0 | 0 | 0 | 0 | 0 | 0 | 0 | 0 | 1 | 1 | 0 | 0 | 0 | 0 | 1 | 1 | 0 | 0 |
| Listening oral definition | 0 | 1 | 1 | 0 | 0 | 0 | 0 | 0 | 0 | 0 | 0 | 0 | 0 | 0 | 0 | 0 | 0 | 0 | 1 | 0 | 0 | 0 | 0 | 1 | 1 | 0 | 0 |
| Naming from definition | 0 | 1 | 1 | 1 | 1 | 0 | 0 | 0 | 0 | 0 | 0 | 0 | 0 | 0 | 0 | 0 | 0 | 0 | 1 | 0 | 0 | 1 | 1 | 1 | 1 | 0 | 0 |
| Picture naming | 1 | 0 | 0 | 1 | 1 | 0 | 0 | 0 | 0 | 0 | 0 | 0 | 0 | 0 | 0 | 0 | 0 | 1 | 1 | 0 | 0 | 1 | 1 | 0 | 0 | 0 | 0 |
| Picture naming with auditory semantic cues | 1 | 1 | 1 | 1 | 1 | 0 | 0 | 0 | 0 | 0 | 0 | 0 | 0 | 0 | 0 | 0 | 0 | 1 | 1 | 0 | 0 | 1 | 1 | 1 | 1 | 0 | 0 |
| Picture naming with error judgment | 1 | 1 | 1 | 1 | 1 | 0 | 0 | 0 | 0 | 0 | 0 | 0 | 0 | 0 | 0 | 0 | 0 | 1 | 1 | 0 | 0 | 1 | 1 | 1 | 1 | 0 | 0 |
| Picture naming with orthographic cues | 1 | 0 | 0 | 1 | 1 | 0 | 0 | 0 | 0 | 1 | 0 | 0 | 0 | 0 | 0 | 0 | 0 | 1 | 1 | 0 | 0 | 1 | 1 | 0 | 0 | 1 | 0 |
| Picture naming with orthographic cues (letter, syllable, word) and distractors words | 1 | 0 | 0 | 1 | 1 | 1 | 1 | 0 | 0 | 1 | 1 | 0 | 0 | 0 | 0 | 0 | 0 | 1 | 1 | 0 | 0 | 1 | 1 | 0 | 0 | 1 | 1 |
| Picture naming with orthographic cues and reading aloud | 1 | 0 | 0 | 1 | 1 | 1 | 0 | 0 | 0 | 1 | 1 | 0 | 0 | 0 | 0 | 0 | 0 | 1 | 1 | 0 | 0 | 1 | 1 | 0 | 0 | 1 | 1 |
| Picture naming with phonological cues | 1 | 0 | 0 | 1 | 1 | 0 | 0 | 0 | 0 | 0 | 0 | 0 | 0 | 1 | 0 | 0 | 0 | 1 | 1 | 0 | 0 | 1 | 1 | 1 | 0 | 0 | 0 |
| Picture naming with phonological cues (letter, syllable, word) and distractors words | 1 | 1 | 1 | 1 | 1 | 0 | 0 | 0 | 0 | 0 | 0 | 0 | 0 | 1 | 0 | 1 | 0 | 1 | 1 | 0 | 0 | 1 | 1 | 1 | 1 | 0 | 0 |
| Picture naming with syllable counting and first phoneme, identification of picture naming errors | 1 | 0 | 0 | 1 | 1 | 0 | 0 | 0 | 0 | 0 | 0 | 0 | 0 | 0 | 0 | 0 | 0 | 1 | 1 | 0 | 0 | 1 | 1 | 0 | 0 | 0 | 0 |
| Picture naming with syllables counting and iconic gesture of the target | 1 | 0 | 0 | 1 | 1 | 0 | 0 | 0 | 0 | 0 | 0 | 0 | 0 | 0 | 0 | 0 | 0 | 1 | 1 | 0 | 0 | 1 | 1 | 0 | 0 | 0 | 0 |
| Picture naming with visual semantic cues | 1 | 0 | 0 | 1 | 1 | 0 | 0 | 0 | 0 | 0 | 0 | 0 | 0 | 0 | 0 | 0 | 0 | 1 | 1 | 0 | 0 | 1 | 1 | 0 | 0 | 0 | 0 |
| Point to initial letter of auditory words | 0 | 0 | 0 | 0 | 0 | 0 | 0 | 1 | 1 | 0 | 0 | 0 | 0 | 0 | 0 | 0 | 0 | 1 | 1 | 1 | 1 | 0 | 0 | 0 | 0 | 0 | 0 |
| Point to initial/final letter of auditory words | 0 | 0 | 0 | 0 | 0 | 0 | 0 | 1 | 1 | 0 | 0 | 0 | 0 | 0 | 0 | 0 | 0 | 1 | 1 | 1 | 1 | 0 | 0 | 0 | 0 | 0 | 0 |

Table 2, continued

| | PA-C | PI-PIL | PIL-C | C-POL | POL-PO | OI-OIL | OIL-C | C-OOL | OOL-OO | OI-PO | OIL-POL | OIL-OOL | OI-OO | PI-PO | PI-OO | PIL-POL | PIL-OOL | PA | C | OOL | OO | POL | PO | PI | PIL | OI | OIL |
|---|---|---|---|---|---|---|---|---|---|---|---|---|---|---|---|---|---|---|---|---|---|---|---|---|---|---|---|
| Point to written word that rhymes with auditory word | 0 | 0 | 0 | 1 | 0 | 1 | 1 | 1 | 1 | 1 | 1 | 0 | 0 | 0 | 0 | 0 | 0 | 1 | 1 | 1 | 1 | 1 | 0 | 0 | 0 | 1 | 1 |
| Question about function of object (pictures) | 0 | 1 | 1 | 1 | 1 | 0 | 0 | 0 | 0 | 0 | 0 | 0 | 0 | 0 | 0 | 0 | 0 | 0 | 1 | 0 | 0 | 1 | 1 | 1 | 1 | 0 | 0 |
| Reading aloud | 0 | 0 | 0 | 1 | 1 | 1 | 1 | 0 | 0 | 1 | 1 | 0 | 0 | 0 | 0 | 0 | 0 | 0 | 1 | 0 | 0 | 1 | 1 | 0 | 0 | 1 | 1 |
| Reading aloud with picture | 1 | 0 | 0 | 1 | 1 | 1 | 1 | 0 | 0 | 1 | 1 | 0 | 0 | 0 | 0 | 0 | 0 | 1 | 1 | 0 | 0 | 1 | 1 | 0 | 0 | 1 | 1 |
| Repetition | 0 | 1 | 1 | 1 | 1 | 0 | 0 | 0 | 0 | 0 | 0 | 0 | 0 | 1 | 0 | 1 | 0 | 0 | 1 | 0 | 0 | 1 | 1 | 1 | 1 | 0 | 0 |
| Repetition with iconic gesture | 0 | 1 | 1 | 1 | 1 | 0 | 0 | 0 | 0 | 0 | 0 | 0 | 0 | 1 | 0 | 1 | 0 | 0 | 1 | 0 | 0 | 1 | 1 | 1 | 1 | 0 | 0 |
| Repetition with picture | 1 | 1 | 1 | 1 | 1 | 0 | 0 | 0 | 0 | 0 | 0 | 0 | 0 | 1 | 0 | 1 | 0 | 1 | 1 | 0 | 0 | 1 | 1 | 1 | 1 | 0 | 0 |
| Semantic judgement on auditory word | 0 | 1 | 1 | 0 | 0 | 0 | 0 | 0 | 0 | 0 | 0 | 0 | 0 | 0 | 0 | 0 | 0 | 1 | 1 | 0 | 0 | 0 | 0 | 1 | 1 | 0 | 0 |
| Simultaneous auditory and written word-picture matching | 1 | 1 | 1 | 0 | 0 | 1 | 1 | 0 | 0 | 0 | 0 | 0 | 0 | 0 | 0 | 0 | 0 | 1 | 1 | 0 | 0 | 0 | 0 | 1 | 1 | 1 | 1 |
| Spoken-to-written phoneme matching | 0 | 0 | 0 | 0 | 0 | 0 | 0 | 0 | 0 | 1 | 0 | 0 | 0 | 1 | 1 | 0 | 0 | 0 | 0 | 0 | 0 | 0 | 1 | 1 | 0 | 1 | 0 |
| Syllable judgement on word supplied with the picture | 1 | 1 | 1 | 0 | 0 | 0 | 0 | 0 | 0 | 0 | 0 | 0 | 0 | 0 | 0 | 0 | 0 | 1 | 1 | 0 | 0 | 0 | 0 | 1 | 1 | 0 | 0 |
| Word length judgment on auditory words | 0 | 1 | 1 | 0 | 0 | 0 | 0 | 0 | 0 | 0 | 0 | 0 | 0 | 0 | 0 | 0 | 0 | 0 | 1 | 0 | 0 | 0 | 0 | 1 | 1 | 0 | 0 |
| Written anagram and reading | 0 | 0 | 0 | 1 | 1 | 1 | 1 | 0 | 0 | 1 | 1 | 0 | 0 | 0 | 0 | 0 | 0 | 0 | 1 | 0 | 0 | 1 | 1 | 0 | 0 | 1 | 1 |
| Written generation of semantic cues of picture | 1 | 0 | 0 | 0 | 0 | 0 | 0 | 1 | 1 | 0 | 0 | 0 | 0 | 0 | 0 | 0 | 0 | 1 | 1 | 1 | 1 | 0 | 0 | 0 | 0 | 0 | 0 |
| Written picture naming | 1 | 0 | 0 | 0 | 0 | 0 | 0 | 1 | 1 | 0 | 0 | 0 | 0 | 0 | 0 | 0 | 0 | 1 | 1 | 1 | 1 | 0 | 0 | 0 | 0 | 0 | 0 |
| Written picture naming with anagram, orthographic cues | 1 | 0 | 0 | 0 | 0 | 1 | 1 | 1 | 1 | 0 | 0 | 0 | 1 | 0 | 0 | 0 | 0 | 1 | 1 | 1 | 1 | 0 | 0 | 0 | 0 | 1 | 1 |
| Written word picture matching | 1 | 0 | 0 | 0 | 0 | 1 | 1 | 0 | 0 | 0 | 0 | 0 | 0 | 0 | 0 | 0 | 0 | 1 | 1 | 0 | 0 | 0 | 0 | 0 | 0 | 1 | 1 |
| Written word picture matching with reading aloud | 1 | 0 | 0 | 1 | 1 | 1 | 1 | 0 | 0 | 1 | 1 | 0 | 0 | 0 | 0 | 0 | 0 | 1 | 1 | 0 | 0 | 1 | 1 | 0 | 0 | 1 | 1 |
| Writting to dictation | 0 | 1 | 1 | 0 | 0 | 0 | 0 | 1 | 1 | 0 | 0 | 0 | 0 | 1 | 1 | 1 | 1 | 0 | 1 | 1 | 1 | 0 | 0 | 1 | 1 | 0 | 0 |

Table 3
Results of the meta-analysis

| | PA-C | PI-PIL | PIL-C | C-POL | POL-PO | OI-OIL | OIL-C | C-OOL | OOL-OO | OI-PO | OIL-POL | OIL-OOL | OI-OO | PI-PO | PI-OO | PIL-POL | PIL-OOL | PA | C | OOL | OO | POL | PO | PI | PIL | OI | OIL |
|---|---|---|---|---|---|---|---|---|---|---|---|---|---|---|---|---|---|---|---|---|---|---|---|---|---|---|---|
| Hit | 98 | 83 | 83 | 97 | 98 | 46 | 52 | 37 | 21 | 45 | 26 | 7 | 6 | 73 | 1 | 63 | 1 | 96 | 106 | 21 | 20 | 98 | 98 | 82 | 82 | 54 | 37 |
| Miss | 12 | 14 | 14 | 12 | 12 | 4 | 4 | 2 | 1 | 2 | 1 | 1 | 1 | 10 | 0 | 10 | 0 | 12 | 17 | 1 | 1 | 12 | 12 | 14 | 14 | 4 | 3 |
| Correct Rejection | 5 | 3 | 3 | 5 | 5 | 13 | 13 | 15 | 16 | 15 | 16 | 16 | 16 | 7 | 17 | 7 | 17 | 5 | 0 | 16 | 16 | 5 | 5 | 3 | 3 | 13 | 14 |
| False Alarm | 9 | 24 | 24 | 10 | 9 | 61 | 55 | 70 | 86 | 62 | 81 | 100 | 101 | 34 | 106 | 44 | 106 | 11 | 1 | 86 | 87 | 9 | 9 | 25 | 25 | 53 | 70 |
| d’ | 0,87 | −0,16 | −0,16 | 0,80 | 0,87 | 0,47 | 0,59 | 0,70 | 0,68 | 0,86 | 0,81 | 0,06 | −0,03 | 0,22 | | 0,00 | | 0,73 | | 0,68 | 0,65 | 0,87 | 0,87 | −0,19 | −0,19 | 0,63 | 0,47 |
| beta | 0,50 | 1,20 | 1,20 | 0,52 | 0,50 | 0,58 | 0,50 | 0,41 | 0,40 | 0,33 | 0,33 | 0,93 | 1,03 | 0,79 | | 1,00 | | 0,53 | | 0,40 | 0,42 | 0,50 | 0,50 | 1,24 | 1,24 | 0,48 | 0,57 |
| mean | 0,72 | −0,10 | −0,08 | 0,90 | 1,25 | 0,55 | 0,49 | 0,62 | 0,62 | 1,10 | 0,73 | −0,18 | −0,23 | 0,42 | | 0,00 | | 0,73 | | 0,53 | 0,50 | 1,22 | 1,24 | −0,16 | −0,18 | 0,58 | 0,53 |
| sd | 0,41 | 0,37 | 0,41 | 0,43 | 0,49 | 0,36 | 0,37 | 0,35 | 0,34 | 0,33 | 0,30 | 0,42 | 0,41 | 0,37 | | 0,35 | | 0,43 | | 0,35 | 0,33 | 0,47 | 0,47 | 0,38 | 0,39 | 0,36 | 0,37 |
| 2.5% quantile | −0,11 | −0,81 | −0,86 | 0,08 | 0,34 | −0,11 | −0,16 | −0,11 | −0,05 | 0,47 | 0,04 | −1,12 | −1,07 | −0,34 | | −0,66 | | −0,11 | | −0,11 | −0,21 | 0,26 | 0,35 | −0,88 | −0,95 | −0,11 | −0,13 |
| 97.5% quantile | 1,49 | 0,63 | 0,71 | 1,78 | 2,30 | 1,26 | 1,19 | 1,20 | 1,26 | 1,75 | 1,23 | 0,51 | 0,44 | 1,13 | | 0,72 | | 1,57 | | 1,17 | 1,14 | 2,21 | 2,20 | 0,57 | 0,56 | 1,25 | 1,25 |
| 0.5% quantile | −0,48 | −0,98 | −0,95 | −0,20 | 0,03 | −0,35 | −0,38 | −0,28 | −0,44 | 0,29 | −0,28 | −1,20 | −1,34 | −0,49 | | −0,79 | | −0,40 | | −0,45 | −0,43 | 0,05 | 0,08 | −0,95 | −1,07 | −0,31 | −0,29 |
| 0.05% quantile | −0,60 | −1,07 | −1,01 | −0,39 | −0,17 | −0,49 | −0,53 | −0,37 | −0,60 | 0,21 | −0,40 | −1,67 | −1,49 | −0,77 | | −0,95 | | −0,55 | | −0,58 | −0,61 | −0,16 | −0,22 | −1,01 | −1,16 | −0,51 | −0,42 |
| *0.05; **0.01; ***0.001 | | | | * | ** | | | | | *** | * | | | | | | | | | | | ** | ** | | | | |

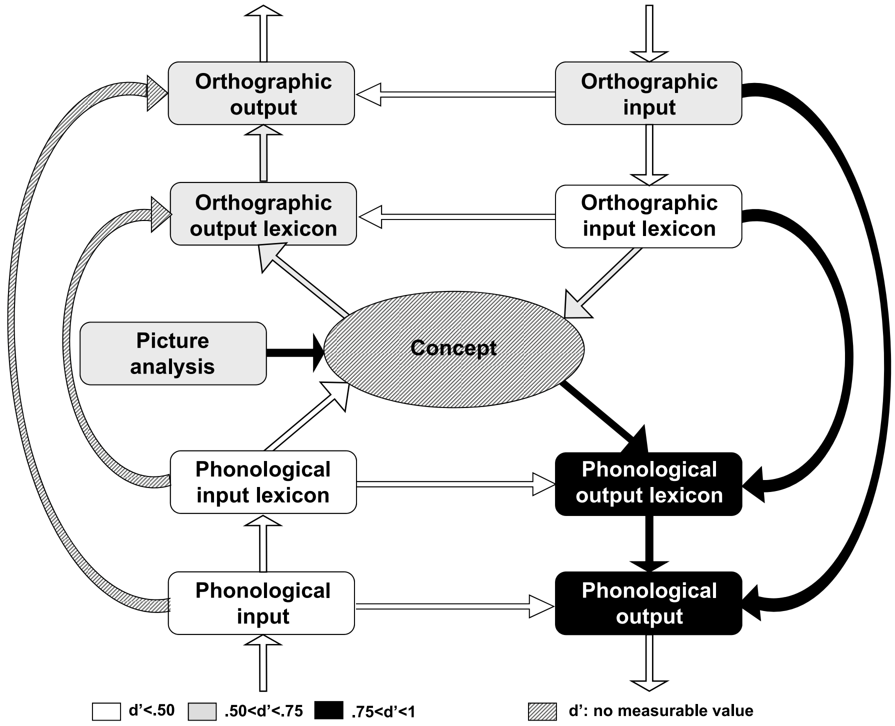

Fig. 2. D' values of the effect of rehabilitation reported on the model of language processing.

the rehabilitation tasks, we scored a FALSE ALARM if the rehabilitation came out positive, and a CORRECT REJECTION otherwise. We then computed d' and beta for each processing component, by using the average HIT and FALSE ALARM rates across all of the case studies [39]. The value of d' can be interpreted as a bias-free measure of the sensitivity of the reeducation strategy, i.e. a measure of rehabilitation effectiveness, and beta as a measure of bias (i.e. the bias to only report or publish rehabilitation case studies that work, or the unspecific effect of rehabilitation). The effect of generalization is not included in our meta-analysis because of the small number of studies in which it was reported. Note that in the meta-analysis, the activation of each of the processing levels and connections were considered individually for computing the effect of therapy. We did not analyse the different combination of processing levels or connections that could jointly affect the effectiveness of therapy. Indeed our model is already quite complex (10 nodes, 17 links), and the number of possible simple combinations between these elements becomes too large (351 combinations) compared to the number of data points available (124 outcomes), especially since none of the published data attempt to rehabilitate the processing levels in systematic or a factorial design.

### *2.3. Results and discussion*

The values of d' and beta are shown in Table 3. The imbalance between the number of case studies in which the rehabilitation benefited to the patients and those with no results ($N = 105$ vs. $N = 19$) result in a large response bias (from 0.33 to 1.24, with an average 0.67) which is rather homogeneous across processing components. This might be interpreted either as a generic effect of rehabilitation (placebo effect) or, alternatively, as a reporting bias. The d' values, which range between $-0.19$ and 0.87 are projected onto the processing model using gray levels in Fig. 2. Interestingly, the level that gave the highest value of d' are the phonological output lexicon (d' $= 0.87$, $p < 0.01$), the output phonology (d' $= 0.87$, $p < 0.01$) and the links between these two levels (d' $= 0.87$, $p < 0.01$). Given that the conceptual level is always trained in all rehabilitation studies, it was impossible to derive a d' score for this level. Yet, the link between the conceptual level and the phonological output lexicon yielded a d' of 0.80 ($p < 0.05$). Noteworthy is the role of the links between input (lexical and sublexical) orthography to the output (lexical and sublexical) phonology (d' $= 0.81$, $p < 0.05$ and d' $= 0.86$, $p < 0.001$, respectively). The other levels had a lower d' which did not reach significance. In particular, the phonological input and the phonological input lexicon had a low d' of $-0.19$.

This meta-analysis shows that all the tasks are not equally efficient in producing a positive effect [5,14,25, 40–42]. Indeed, rehabilitation of the phonological output lexicon and of the phonological output of the word production levels are more prone to successful rehabilitation than, say, the phonological input components or the components of the orthographic modality. The relative inefficiency of the phonological input components is interesting to consider given the widespread use of syllable counting, monitoring techniques and phonological cueing techniques in rehabilitation studies [5, 10,12,18,20,25,26,43–52]. Surprisingly, word production can also benefit from rehabilitation triggering the pathways linking orthographic input to phonological output and orthographic input lexicon to phonological output lexicon. This may result from an alternative strategy induced by reading aloud words and pseudowords using grapheme-phoneme direct conversion; patients that are trained in reading tasks may produce a word by accessing the orthographic form of the word to be named, and producing it aloud.

This set of results shows that the use of a processing model can help to extract more information from rehabilitation studies than can be done in a cumulative survey, even when the rehabilitation tasks have not been specifically designed to be specific to a particular processing component. This conclusion is similar to that reached by Indefrey and Levelt [53] with a similar methodology applied to functional imagery.

Yet, we should be aware that this kind of meta-analysis is inherently limited in four ways: it is dependant on the quality of the patient's diagnostic, on the adequacy of the processing model, on the adequacy of the modeling of each of the tasks, and on the correlations within the data set itself. First, given that many of the published rehabilitation studies we used in this meta-analysis did not report any detailed diagnostic test to the specific locus of patient impairment, (no attempt to specify brain localization), this information could not be taken into account in our meta-analysis. Yet, it is likely that rehabilitation outcomes may vary with the specific pattern of production impairment. Second, the processing model does not distinguish between different subcomponents of lexical processing, yet these components could be differentially targeted by rehabilitation strategies. Third, we included in the analysis only the standard pathways for performing a given task, yet, different patients may use differing strategies by employing alternate pathways to perform a given task. Fourth, the published studies did not independently target each possible rehabilitation foci (or combination of foci). While the signal detection analysis can take into account the relative frequencies of these rehabilitation strategies, nothing can be done when a particular focus or combination is missing. For instance, the role of the semantic/conceptual component cannot be evaluated statistically because all of the studies included a task that involved this component; hence there cannot be any observation in the FALSE ALARMS and CORRECT REJECTION cells. This can only be alleviated by conducting a rehabilitation study that manipulates this component. Another example is given by the fact that in virtually all of the studies, the phonological output lexicon, the phonological output and the connection between these two levels are either all simultaneously active or inactive. It is therefore impossible to disentangle the role of these various components in the meta-analysis alone. These limitations make it impossible to dissociate the effects of so-called semantic therapy and phonological therapy within the existing dataset. The same limit would apply if we were to study the effect of the combination of rehabilitation factors: not all such combinations have been tried in our database.

Despite these limitations, our meta-analysis resulted in a picture that was considerably clearer than what a rapid browse of Table 1 suggests. In addition, it is possible to address some of the outstanding issues through a targeted rehabilitation study. In the second part of the paper we therefore establish a specific rehabilitation protocol for an anomic patient in order to assess in a more controlled way the impact of the activation of the different levels (conceptual knowledge, phonological output lexicon, phonological output and phonological input) of word production.

## 3. Case study: DPI

DPI is a 68-year-old, right-handed, retired medical doctor. Five years before the rehabilitation program, he had a stroke leading to Wernicke's aphasia and a right hemiparesis. A CT scan (at admission) and a MRI (one year later) confirmed a left middle cerebral artery stroke extended to the junction territory with the posterior cerebral artery. The lesion encompassed the left temporal artery territory including the superior, medial, and inferior temporal gyri the anterior temporal lobe. A previous detailed study of the patient showed that he suffered from a phonological output lexicon deficit and had preserved conceptual abilities [54]. Briefly,

DPI was flawless in all non verbal tasks that tap conceptual processes using five tasks constructed along the lines of Caramazza and Shelton [55] (anomaly detection, picture completion, intruder detection, functional matching task and object color matching task). On a picture naming task, DPI produced many errors (47.1% of errors, $N = 70$). Because this deficit occurred in the presence of intact conceptual knowledge, and good performance in word reading (10% of errors, $N = 100$), this suggests a deficit at phonological output lexicon. Speech perception was flawless. After the completion of this assessment, the patient suffered a traffic accident resulting in a dramatic deterioration of his word production. DPI received out-clinic speech therapy twice a week. After 12 months, he still complained that he did not recover his former speech production level. For this reason, we proposed to enroll him in a rehabilitation study. We first assessed his performance in naming production, reading and conceptual knowledge in order to ensure that DPI still suffered from a phonological output lexicon deficit. His performance in naming was lower than before (68% of errors, circumlocution or non-responses, $N = 109$) whereas his conceptual knowledge was still intact (errorless performance on the five previous conceptual knowledge tasks), his word comprehension as well (errorless performance on an auditory word – picture matching task, $N = 48$) and his word reading performance remain unchanged (10% of errors). For the duration of our study, the out-clinic speech therapy was suspended.

### *3.1. Method*

We designed the rehabilitation program in order to investigate three issues concerning the rehabilitation of an anomic patient. Firstly, we wanted to further the results of the meta-analysis by determining if the activation of the conceptual knowledge could induce any improvement in word production. Secondly, we wanted to assess whether the activation of the impaired level could be more efficient compared to the activation of non-impaired level within the two levels of the word production system – phonological output lexicon or phonological output. Finally, tasks involving the activation of the phonological input have been extensively utilized in rehabilitation programs, but the results of the meta-analysis suggest that they do not impact positively the word production performance. Therefore, we wanted to prospectively verify whether they could be successful in word production rehabilitation. Thus, DPI was submitted to 4 phases of rehabilitation, each of them involving the activation of a specific speech level: conceptual knowledge, phonological output lexicon and phonological output, and phonological input (Fig. 3). We followed the methodology proposed by Nickels [4]:

- Use of a pre-therapy baseline that should be assessed on more than one occasion to establish degree of spontaneous recovery and/or variability,
- Use of identical sets in terms of naming difficulty for the different rehabilitation phases,
- Use of the same task for assessing patient's performance before and after therapy,
- Use of a task that contains enough items to allow change to be demonstrated,
- Use of statistical comparisons such as McNemar's Test to identify whether any change in performance is greater than might be expected by chance.

#### *3.1.1. Baseline of performance*

To demonstrate the efficacy of a rehabilitation program, it is important to assess whether trained items are better named after the rehabilitation than they were before, thus comparing the magnitude of change attributable to spontaneous recovery and the magnitude of change attributable to therapy. Whereas comparing performance of different groups of patients is marred by the great variability in individual patients' profiles [56, 57], a possible alternative to override the heterogeneity between groups is to conduct a single case study. It consists in comparing performance of a single patient after and before the rehabilitation. Performance baseline is defined before beginning the rehabilitation program and constitutes the control performance as opposed to performance after intervention. This baseline is generally evaluated by testing the patient several times on the same set of items and by quantifying his spontaneous performance variability [4,7,10]. Here, we established the performance baseline by asking DPI to name the same set of pictures three different times. 156 pictures were tested and no feedback was provided. The three successive sessions were separated by two weeks. These sessions were used to quantify DPI's performance variability and constitute the three pretests.

#### *3.1.2. Selection of the picture stimuli*

Among the 156 pictures presented in pretests, we selected the pictures never correctly named. We obtained 106 pictures that were divided in four experimental sets of equal difficulty. The sets were composed of 27 pic-

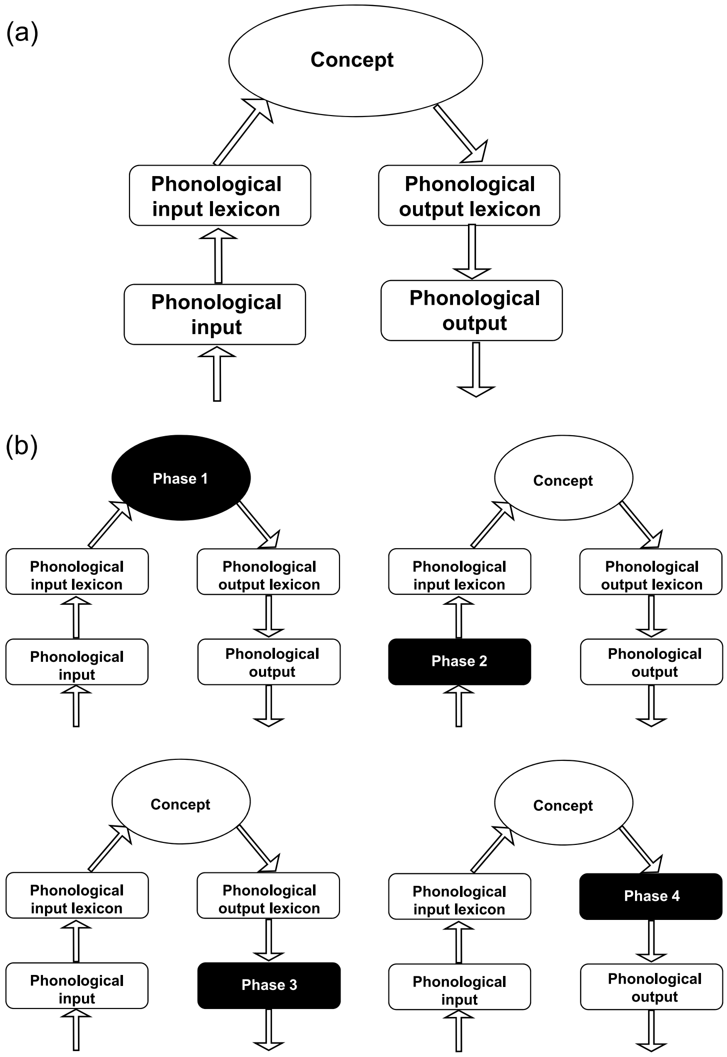

Fig. 3. (a) Simplified model of word processing from Fig. 1. This model includes word comprehension and word production. (b) Levels activated through each rehabilitation phase.

tures (Set 1) or 26 pictures (sets 2, 3 and 4) matched for word frequency, word length, word gender and semantic categories (approximately 50% of pictures depicted artifacts, 19% vegetables, 27% animals, and 4% body parts).

### *3.1.3. Rehabilitation procedure*

The four following levels of word processing were trained during four separate phases (see Fig. 3): conceptual knowledge, phonological input, phonological output and phonological output lexicon. For each phase, we used one of the 4 experimental sets of pictures to construct the training material. The 4 sets were randomly assigned to the 4 rehabilitation phases. Feedback and correct responses were provided to the patient during the rehabilitation phases.

#### *Phase 1: Conceptual knowledge rehabilitation*

Conceptual knowledge corresponds to a non-verbal general knowledge of the world. Several non-auditory tasks involving picture comprehension and conceptual knowledge processing were proposed: anomalous/non anomalous picture categorization, picture completion, categorical intruder detection, functional matching, correct color detection, picture categorization in several semantic categories, and knowledge assessment (see [55]). Details of the procedure are provided in the Supplementary Material section. In all these tasks, only yes/no responses or pointing were expected (ex-

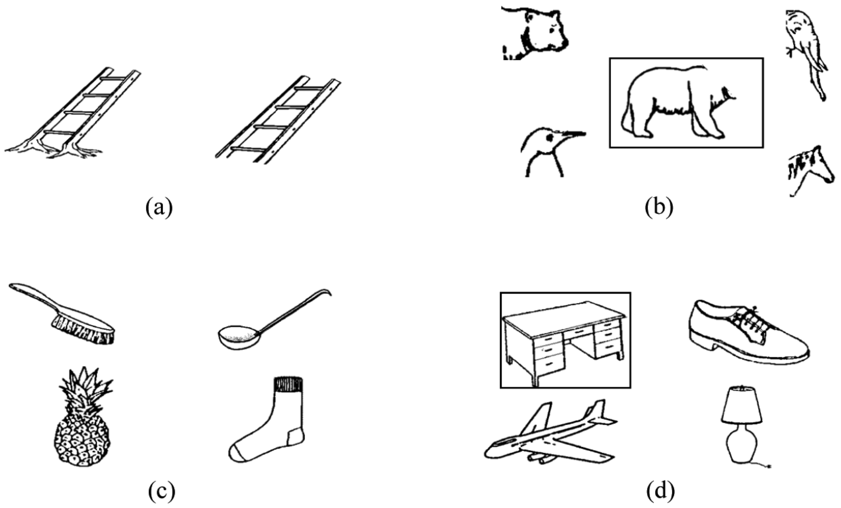

Fig. 4. (a) Example of anomalous and correct pictures, (b) example of picture completion task, (c) example of intruder detection task, (d) example of functional matching task.

amples are provided in Fig. 4). DPI was instructed to never name the picture, neither did the examiner.

*Phase 2: Phonological input rehabilitation*

To avoid access of semantic information related to the picture names of the Set 2, pseudowords were constructed using the speech-sound of the picture names of the Set 2. Three lists of pseudowords were created in order to prevent boredom and effective pseudoword learning. The first list was constructed by intra-word syllables cross-placing in multisyllabic words (e.g. "artichaut" /aRtiʃo/ (artichoke) yielded to /ʃotiaR/) and inter-word phonemes cross-placing in monosyllable words. Therefore, the number of syllables was similar for pseudoword and real word. The second list was constructed by syllable cross-placing inter-word and by preserving the rime of the source words (e.g; "artichaut" and "cerise" /səRiz/ (cherry) yielded to /səʃo/ and /aRtiRiz/). The third list was constructed by inter-word phonemes cross-placing. This list contained exactly the same phonemes and preserves the distribution of mono-, bi-, tri- and quadrisyllables from the original list (e.g.: /ʃəsoRiz/ and /tiaR/ for "artichaud" and "cerise"). The examiner pronounced the pseudoword at random and DPI was asked to perform several tasks involving speech-sound analysis such as syllable, rhyme, phoneme discrimination and detection tasks, auditory-written syllable matching task on syllable and rhyme, and pseudoword and syllable counting. Details are provided in the Appendix section. Again, only yes-no responses and finger pointing were required.

*Phase 3: Phonological output rehabilitation*

This phase was based on speech-sound production. In order to reduce activation of both conceptual knowledge and phonological output lexicon levels we used a type of rebus. The rebus we used consisted of pictorial symbols that represent syllabic or phonemic sounds. Each picture name of Set 3 was decomposed in syllables (except for mono-syllables) and each syllable or phoneme was represented by a pictorial symbol that would elicit the corresponding syllable or phoneme sound, for instance the sound [ki:] was elicited with a picture of a key and so on. The patient was asked to produce the speech-sound corresponding to the pictorial symbol (see Fig. 5). The pictorial symbols were presented either in isolation to make the patient produce monosyllables or diversely associated to make the patient produce pseudowords of increasing complexity. Details are provided in the Appendix section. For the monosyllabic words, the picture used was not the target picture but another picture that elicited the same speech-sound. For instance, the word "poêle" /pwal/ frying pan was instantiated by a picture of a "poil" /pw-al/ hair. For the 54 syllables issued from the picture names of the Set 3, we used a total of 22 pictorial symbols. Our aim was to induce an automatic link between a pictorial symbol and a speech-sound and then to trigger the activation of phonological output via the most direct means, preventing the activation of the other levels involved in word production. Of course, the use of abstract symbol might have been methodologically purer, but would have been required DPI to learn to associate a given abstract symbol and a speech-sound,

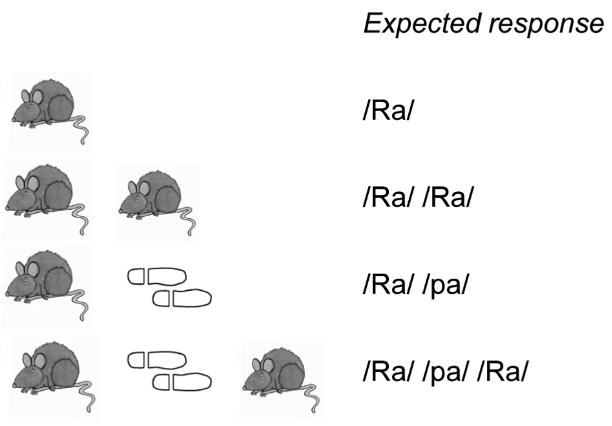

Fig. 5. Example of rebus for syllables (Rat in French is pronounced /Ra/ and step /pa/).

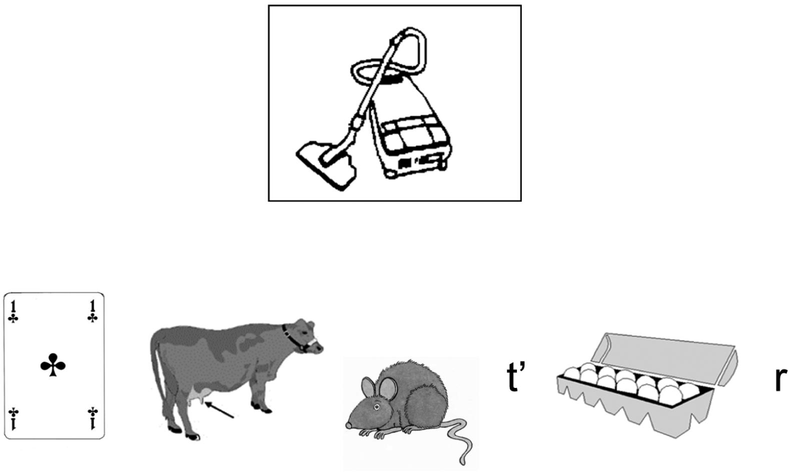

Fig. 6. Example of the naming task with the picture and the rebus for syllables. Vacuum cleaner is /as/ /pi/ /ra/ /tœr/ in French.

which was deemed to be too difficult. The patient was always helped when he could not find the associated sound to a given pictorial symbol and rapidly became familiar with them.

*Phase 4: Phonological output lexicon rehabilitation*

Four different strategies could be used to activate the phonological output lexicon: through the repetition of a perceived word, through the reading of a written word, through the production of speech output by the use of a verbal fluency task or a naming task. In this rehabilitation phase, activation of the speech perception system and of the reading system was avoided. Moreover we wanted the patient to activate specific phonological word-forms, the ones that composed the picture names of the Set 4. It is in theory hardly conceivable to activate the phonological output lexicon where the phonological word-forms are retrieved without activating the conceptual knowledge and the phonological output associated to these word-forms. Therefore, we decided to use a naming task that would activate the conceptual level, the phonological output lexicon and the phonological output and to subtract the effect of the activation of the conceptual level obtained during the Phase 1 and the effect of the activation of the phonological output obtained during the Phase 3. Thus, DPI was instructed to name the pictures of the Set 4. Together with the picture, the pictorial symbols corresponding to the syllables of the picture name were presented to DPI. As in the previous phase, each syllable was symbolized by a pictorial symbol, and the pictorial symbols were presented below the target picture (Fig. 6).

*3.1.4. Testing session*

At the end of each rehabilitation phase, we ran a testing session. We used a cross-over design for assessing

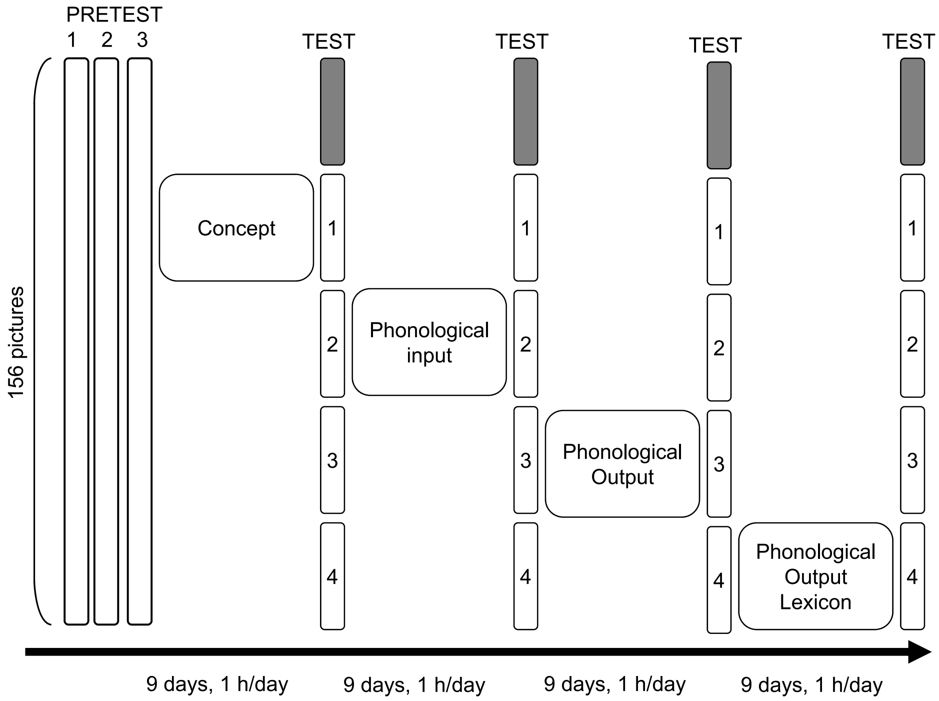

Fig. 7. Flow chart or rehabilitation program. In the pretest, grey rectangles represent the set of items correctly named and white rectangles represent the four sets of unsuccessfully-name items.

DPI's performance. After each rehabilitation phase, DPI was asked to name the all 156 pictures without any feedback. This included the pictures of the set used in a given rehabilitation phase i.e. the trained stimuli, plus the pictures of the three other experimental sets i.e. the untrained stimuli, plus the pictures that the patient had successfully named during the pretests and that were not included rehabilitation material. These last pictures were used to check for spontaneous variability.

The examiner who evaluated the patient's performance in the test sessions was blind to the assignment of the pictures in the 4 experimental sets. Finally, patient and relatives were interviewed in an informal manner about their subjective feelings regarding the rehabilitation program.

#### *3.1.5. Rehabilitation flow-chart*

Because Hillis (1998, see also [58]) has shown that short-term but intensive training program (5 days per week during 2 weeks) induces better improvement than long term non-intensive training program (2 days per week during 5 weeks), our patient was trained daily accordingly. The duration of each rehabilitation phase was of two weeks (Fig. 7). Training was provided one hour per day except on weekends. Each rehabilitation phase was followed by one to two weeks of rest.

### *3.2. Results*

Statistical analysis was performed with the McNemar test [4,10,12] to compare DPI's results before and after the different rehabilitation phases. Three types of analyses were conducted:

Performance after each pretest and after each of the four rehabilitation phases was assessed in all pictures in order to assess whether the rehabilitation program is successful and determine which phase is the most successful;

Performance in each trained set (material set of a rehabilitation phase) before and after the rehabilitation phase was assessed in order to investigate whether trained items were successfully named at the end of the phase. Note that each trained set was different for each rehabilitation phase, ie Set 1 was the trained set for the Phase 1, Set 2 was the trained set of the Phase 2 and so on.

Performance for untrained sets (that is, for each phase, the 3 sets that were not used as trained set during the rehabilitation phase) before and after the rehabilitation phase was assessed in order to investigate whether the successful effect of a phase is generalized to untrained picture names. Note that untrained sets were

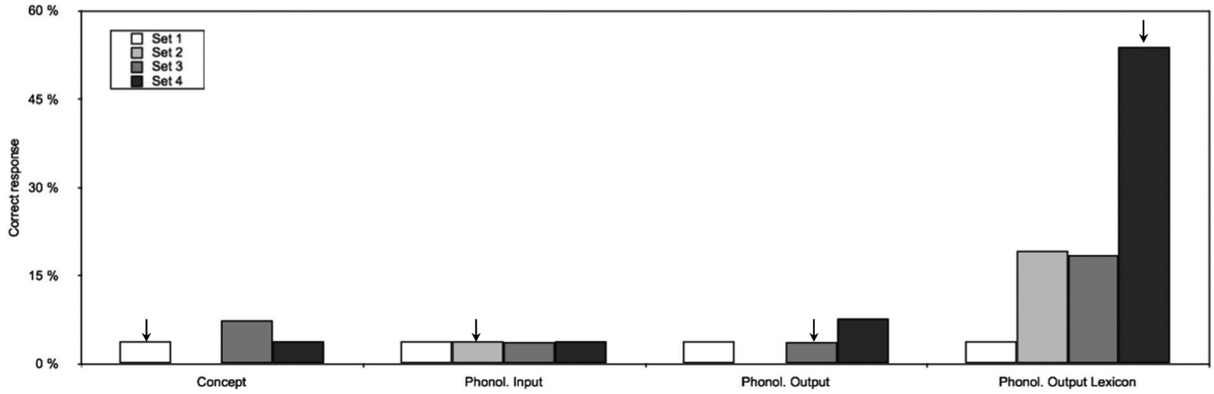

Fig. 8. Naming performance for each subset after the four rehabilitation phases. Arrows indicate the trained set for each rehabilitation phase.

different for each rehabilitation phase, ie sets 2,3,4 were the untrained sets for the Phase 1, sets 1,3,4 were the untrained sets of the Phase 2 and so on.

### 3.2.1. *Global performance: All pictures* ($N = 156$)

The analyses were conducted with all the 156 pictures. This includes the pictures that were successfully named during the pretests ($N = 52$), the pictures that composed the trained set for each rehabilitation phase ($N = 26$–27) and the pictures that composed the untrained set for each rehabilitation phase ($N = 79$–80). In the pretests, performance was remarkably stable (Pretest 3: 42/156 correct vs Pretest 1: 37/156 correct; McNemar $\chi^2 = 0.9$, $p = 0.33$). Comparison of results before and after the rehabilitation program shows a significant effect of the rehabilitation (Phase 4 of rehabilitation: 64/156 correct vs Pretest 3: 42/156; McNemar $\chi^2 = 15.8$, $p < 0.001$). When comparing the results before and after each phase of rehabilitation, performance does not improve after the first three phases (Phase 1: 39/156 correct, Phase 2: 37/156 correct, Phase 3: 39/156 correct; McNemar $\chi^2 < 1, p > 0.1$) but improves after the Phase 4 (Phase 4: 64/156 correct; McNemar $\chi^2 = 17.36, p < 0.001$).

Performance for the successfully named pictures ($N = 52$) was quite accurate and constant along the different phase (Pretest 1: 37/52 correct, Pretest 2: 39/52 correct, Pretest 3: 42/52 correct, Phase 1: 35/52 correct, Phase 2: 33/52 correct, Phase 3: 35/52 correct, Phase 4: 39/52 correct).

*Trained set performance*

The analyses were conducted considering the 26 or 27 pictures of each trained set. Detailed results are provided in Fig. 8. Performance remains stable after the first three rehabilitation phases (McNemar $\chi^2 < 1, p > 0.1$) whereas it dramatically improves after the Phase 4 (Set 4: 16/26 correct vs 2/26 correct; McNemar $\chi^2 = 11, p < 0.001$).

### 3.2.2. *Untrained sets and generalization*

Performance for the untrained sets does not improve after the first three phases (McNemar $\chi^2 < 1, p > 0.1$) whereas it improves after the Phase 4 (Set $1 + 2 + 3$: 13.9% correct vs. 2.5%; McNemar $\chi^2 = 7.11$ $p = 0.007$).

A post-hoc analysis of performance in the untrained sets after the Phase 4 was performed according to the semantic category. Performance improved after the Phase 4 in the artifact category (see Table 4, McNemar $\chi^2 = 5$; $p = 0.025$) but not for the other semantic categories (animals, vegetables and body parts, McNemar $\chi^2 < 1, p > 0.1$).

Finally, DPI reported that he was more confident in speaking with others. This informal evaluation suggests a positive impact of the rehabilitation program on everyday life.

## 3.3. *Discussion*

We conducted a short and intensive rehabilitation program with a stable long standing aphasic patient, DPI. His deficit has been detailed in a previous study and is localized at the phonological output lexicon [54]. Four different phases of rehabilitation that successively activate conceptual knowledge, phonological input, phonological output, and the phonological output lexicon were tested.

DPI's global production performance improved at the end of the rehabilitation program. This confirms that an intensive rehabilitation program can have a significantly positive effect on patient's production perfor-

Table 4
Distribution of correct responses of untrained items according to their semantic category before and after the Phase 4

| | Before | After |
|---|---|---|
| Artifact ($N = 40$) | 1 (2.5%) | 6 (25%) |
| Animal ($N = 20$) | 0 (0%) | 1 (5%) |
| Vegetable ($N = 14$) | 0 (0%) | 2 (14%) |
| Body-part ($N = 5$) | 1 (20%) | 2 (40%) |

mance long after his brain lesion [4,7,59]. Although the benefits of rehabilitation appeared only at the last phase, it is unlikely that they appear solely by practice effects. Practice effects would have induced a gradual increase of patient performance along the four phases rather than an effect restricted to the phase 4. Moreover, before being enrolled in the rehabilitation program, the patient was seeing a speech therapist two times a week with no effect of his speech production performance. For these two reasons, it seems reasonable to attribute the positive outcome after the forth phase essentially to the treatment occurring during this phase.

This experimental data confirm that all tasks are not equivalent in therapy. Specifically, the Phase 1 that induces the activation of the conceptual knowledge did not positively affect the patient production performance, suggesting that the activation of the conceptual level per se is not efficient in terms of speech rehabilitation. Within the word production pathway, two types of rehabilitation were tested. The first one selectively activated the phonological output procedures (phonological planning, articulation), in the absence of lexical processes. The result showed no improvement in the patient's performance. The second one activated the phonological output lexicon which is the impaired level in DPI. Results showed significant improvement of DPI's production performance, meaning that for rehabilitating the production of lexical forms, the more efficient way is to train the patient to produce the words and not only the components of these words. Finally, the focused rehabilitation of input phonology did not yield any improvement confirming that the activation of unimpaired processes, namely input and output phonology did not induce any positive effect of DPI's speech production performance. These experimental results validated and clarified the meta-analysis results showing that rehabilitation tasks that specifically tap into the damaged level, i.e., word production, could make the patient performance in production improve. Of course, the rehabilitation phase that worked was also a phase involving multiple levels, i.e., concept activation, lexical activation and phonological output. Could it be that this aspect of the task was responsible, at least in part, for the results? It is difficult to completely discard this hypothesis, but is it worthwhile mentioning that all of the rehabilitation phases included multiple levels: for instance, the conceptual phase required both picture analysis and conceptual systems, as well as working memory and executive functions. Involving multiple levels can therefore not be the only explanation to the present results.

Rehabilitation is proposed to improve the patient's everyday life, and an important issue with rehabilitation programs is to assess whether performance improvement generalizes to untrained items or whether it is restricted to trained items during the rehabilitation program. Here, the positive effect of Phase 4 is not restricted to the trained set of items but spreads to encompass some other untrained pictures. In previous papers, generalization is inconsistently observed and the factors that could explain this variability are still unknown. It has been proposed that the generalization may be driven by semantic factors. Indeed, Miceli and collaborators [10] described two patients with phonological output lexicon deficit who take significantly advantage from speech rehabilitation but only to name trained items. They proposed that generalization may occur when untrained items are semantically related to the trained ones. In our study, pictures were selected from four semantic categories: animal, body-part, artifact and vegetable and these four categories were equally distributed within the four sets. If any effect of semantic priming is expected, one could predict that it would be greater for items belonging to a homogenous category, as for instance, the body-part category. Considering the four categories in our study, the artifact category is the largest one and the most diversified. It ranges from wall to paperclip, from cigarette to skirt. Thereby, it's unlikely that items from artifact category would prime the other items of this category because of their semantic content distance. Contrary to this prediction, after the Phase 4, our results show that for the untrained sets, the performance improvement is only significant for items from the artifact category (Table 4). This suggests that generalization does not result from an effect of facilitation for semantically related items. What other factors could be proposed to explain the generalization of performance to untrained items? In addition to the semantic properties of the items, generalization may also depend on the mechanisms that promote word recovery. Indeed, there are at least two mechanisms that could offer plausible explanations for the positive outcome of a rehabilitation program. They are (i) the restoration of the damaged level and (ii) the development of compensatory strategies that allow the damaged level to be bypassed. Generalization, accordingly, may be differently affected. Finally, it could be the case that generalization depends on the type of impairment. DPI's impairment involves the phonological output lexicon, but the damage he sustained could impact the access to this level or this level itself. Rehabilitation may differently affect the connection between levels as well as the levels themselves. Hence, factors that favored generalization still need to be explored.

## 4. General discussion

In order to address the specificity of rehabilitation strategies for aphasic patients, our approach was to firstly provide a comprehensive meta-analysis of the relevant peer-reviewed literature on word production rehabilitation. We first introduced a functional model of language processing (Fig. 1). Based on this model, we conducted a functional reconstruction meta-analysis of 39 studies involving a total of 124 rehabilitation cases. Our technique, inspired by model-based meta-analyses of fMRI data [32], consists of dissecting the tasks used in rehabilitation in terms of the processing components of the model, and subsequently in reconstructing the contribution of each component to the rehabilitation outcome. The enterprise of relating the effect of speech rehabilitation to the activated components of spoken and written word processing requires a detailed, explicit theory of the process of word processing. The meta-analysis presented here is based on a consensual model of language processing [28,60]. This model explicates the successive computational stages of spoken and written word perception and spoken and written word production. The componential analysis of the tasks involved in the different rehabilitation studies provided the processes and pathways involved in each of the tasks. The results of the meta-analysis, however, do not hinge on this particular choice of theory, since differences between this model and other models [30,32,61–65] do not concern the assumed processing levels but the exact nature of the information flow between them. The meta-analysis provides a clear-cut picture, wherein only the phonological output processing components (phonological output lexicon and output phonology) significantly contribute to rehabilitation success.

Secondly, we experimentally tested this outcome with a case rehabilitation study on a patient specifically impaired in the phonological lexicon. We used four sets of 'pure' tasks that specifically target one of the following: conceptual knowledge, input phonology, output phonology, or the phonological output lexicon. Only the latter rehabilitation tasks yielded any improvement for the patient, confirming the above conclusion regarding specificity of rehabilitation procedures. Furthermore, this successfully trained processing component was able to generate significant improvement on untrained items, thereby displaying generalization. This result supports the claim that the specific component yielding rehabilitation success was precisely the component that was impaired in this patient (the phonological output lexicon). Yet, because the phonological output lexicon is deeply embedded within the global language model, it is impossible to train this component alone, that is, without simultaneously involving other components that function as inputs or outputs. Our conclusions rest on the fact that independent training of these other components did not improve patient's performance (see Fig. 3). However, we recognize that the successful rehabilitation task was also the only one that encompassed multiple components of speech production processing. We therefore cannot discard that the positive effect of the phonological output lexicon activation is not specifically due to the phonological output lexicon component per se, but due to the fact that the training task involved a processing chain linking the phonological output lexicon to its normal input and output in the speech production pathway.

Overall, the outcome of this study confirms that all therapies are not equally effective and that a rehabilitation focused on his deficit could partially reactivate the impaired process. Even if this idea motivated many past studies [2,4,14,16,49,66], the previous literature of rehabilitation studies failed to reach such conclusion. Furthermore, our data reinforce the importance of a model-based approach for specifying the components impaired in the patient, as well as the tasks used for rehabilitation (see [4]. As many rehabilitation studies did not report the specific locus of patient impairment, we were not able to take into account this information in our meta-analysis. Thus, the next step of this type of analysis would be to include the locus of the deficit and to correlate it with the rehabilitation outcome as we did for the case study, using the same methodology.

From a clinical point of view, this data could help therapists in developing rehabilitation tools for aphasic patients. Speech production deficit typically could involve one or several components. If our results generalize to these other components, a patient-specific rehabilitation strategy, focussing on the impaired components and their connections with the rest of the system could prove more time-effective than generic rehabilitation. Of course, further work is needed in order to assess whether our conclusions hold up quantitatively with more patients, and across different kinds of aphasic deficits.

Two directions in particular would be worth exploring. First, as presented in the introduction, word production problems can surface with two distinct profiles: (1) patients with predominantly semantic paraphasia, who can be described as having an impaired link between the conceptual component and the phonological lexicon, (2) patients with predominantly phonological paraphasia, who have a deficit located at the phonological lexicon (or in the link with the phonological representation) [61,63]. It would be very interesting to use our approach to refine the so-called 'semantic' and 'phonological' rehabilitation but using purer tasks, and to test whether the most successful rehabilitation strategies are indeed the ones linked to the impaired components. A second direction of research is inspired by the functional model itself. Such a model contains many parallel and partially redundant routes. A given task can therefore be performed using several more or less efficient strategies. For instance, to perform a picture naming task, instead of using the phonological output lexicon, one could covertly recover the spelling of the word from the orthographic output lexicon, and then use spelling-to-sound conversion to generate a phonological output. In our meta-analysis (see Fig. 2), we see that, indeed, the spelling-to-sound route has a positive impact on rehabilitation outcome, suggesting that such backup strategies could be useful to incorporate into a complete rehabilitation procedure. Of course, the contribution of these alternate strategies would have to be assessed independently of the direct rehabilitation of the impaired component.

Finally, most of the rehabilitation studies contained very few details regarding the anatomy of the lesions, so it was not possible to integrate anatomical information into the meta-analysis. However, such an approach could benefit from the analysis of the brain regions involved in the deficit and/or the recovery. It would be, in principle, possible to apply our signal detection approach using intact versus lesioned brain regions as input to the analysis [67,68]. Additionally, functional imaging data, coupled with an anatomo-functional processing model could enable to study the effect of different rehabilitation strategies (see [69,70]. In order to enable this kind of study, much more effort towards normalization and systematic archiving of patient's 3D anatomical and functional imaging data is needed (see [71] for an initiative for functional imaging).

## 5. Appendix: Rehabilitation tasks

### *5.1. Conceptual knowledge*

*1. Anomalous/non anomalous picture categorization*

For each picture a corresponding anomalous plausible picture was drawn (Fig. 4, a). DPI was asked to categorize the anomalous and the normal pictures separately and to indicate the anomalous part of the picture.

*2. Picture completion*

A part of each picture was printed and the subject was asked to complete it with one part among four pieces of drawing belonging to different pictures. To sensitize the task and make it non-perceptual but only semantic, the picture parts were not perceptually complementary and their orientation and size were modified (Fig. 4, b).

*3. Categorical intruder detection*

A intruder detection tasks was constructed using each of the target picture. Each picture was presented, at random location, on a sheet of papers with 3 other pictures belonging to a another semantic category. DPI was asked to detect the intruder picture (Fig. 4, c).

*4. Functional matching (for artifacts)*

A multiple-choice task was constructed containing the target picture, a functional related picture and two distracters. DPI was asked to point out the functional related picture. Because functionality refers mostly to artifacts, this experiment was conducted only with artifacts (Fig. 4, d).

*5. Correct color detection (for vegetables)*

A multiple-choice task was constructed using each picture of vegetables. Each vegetable was presented in four exemplars: one with its correct color, and 3 exemplars of incorrect color in random position. DPI was instructed to point to the correct colored picture.

*6. Picture categorization*

DPI was instructed to categorize the pictures in four semantic categories.

*7. Knowledge assessment*

Thirteen type of questions requiring a yes/no response were asked about the items displayed in the pictures: for example "Is it eatable?", "Can it be put in a shoe box?", "Does it live in France?", "Does it have seeds? " (for vegetables), "How to use it?" (for artifacts), "How many legs does it have?" (for animals) etc.

### 5.2. Phonological input

*1. Rhyme judgement*

The examiner pronounced two pseudowords which rhymed or did not rhyme and DPI was asked to say if the two pseudowords rhymed or not (e.g. banoume and panoume, expected response: yes).

*2. Discrimination task*

The examiner pronounced two pseudowords that could be the same or not (if not they differed by a single phoneme) and DPI had to decide if the two pseudowords were or not identical (e.g. banoume and panoume, expected response: no). Another version of this task consisted in repeating three or four times the first pseudoword before pronouncing the second one.

*3. Phoneme detection*

A target vowel or consonant was presented to DPI (auditory and written modality) and he had to indicate if it was contained in the pseudoword pronounced by the examiner (e.g. Is the sound 'v' in fanre, expected response: no).

*4. Syllable detection*

A target syllable was presented to DPI (auditory and written modality) and he had to indicate if it was contained in the pseudoword pronounced by the examiner (e.g. Is the sound 'vo' in volire, expected response: yes).

*5. Syllable number identification*

DPI had to indicate the number of syllables composing the pseudowords pronounced by the examiner. DPI could respond orally or by pointing the correct number written on a sheet of paper.

*6. Auditory written syllable matching*

DPI had to point the written syllable corresponding to the auditory syllable pronounced by the examiner. The target written syllable was presented among a choice of 3, 6 or 12 syllables. (e.g. The sound 'vo' has to be matched with his written form among for instance 3 possibilities vo, fo and ka).

*7. Auditory written pseudoword matching*

DPI had to point the written pseudoword corresponding to the auditory syllable pronounced by the examiner. The target written pseudoword was presented among a choice of 3, 6 or 12 syllables.

*8. Auditory written rhyme matching*

DPI had to point the written rhyme corresponding to the auditory rhyme pronounced by the examiner. The target written rhyme was presented among a choice of 3, 6 or 12 syllables (e.g. the item 'volire' has to be matched with the written item that would rhyme with 'volire' if pronounced among for instance 3 possibilities: ire, are, and ile).

### 5.3. Phonological output

*1. Production of single syllables*

DPI had to pronounce the sounds represented by the rebus. 22 rebus were used to illustrate the all set of syllables. As the aim of this session was to make DPI produce speech sounds, if DPI had difficulties in finding the speech sound corresponding to a given rebus, the examiner pronounced the name of the rebus.

*2. Production of syllabic sequences with increasing complexity*

To make DPI produce several syllables, more than one rebus was presented to him. We began to present a repetition of the same syllable and then complicating the task, we presented different syllables gradually increasing the number of different syllables (Fig. 5).

*3. Production of syllable with various rhythms*

Rebuses were presented with some indications of rhythm. Under rebus a white circle indicated that DPI had to make a "long" syllable, a black circle a normal syllable and an hyphen stood for silence.

## Acknowledgements

The authors thank J. Belmihoub, M. Chevalier, O. Decouche and M. Magné for their help with this work. The current study was supported by Direction Régionale de la Recherche Clinique (AP/HP) through a PRA grant and by a contract interface INSERM AP/HP awarded to AC Bachoud-Lévi.
